# Response to: Comment on “Responders to Platelet-Rich Plasma in Osteoarthritis: A Technical Analysis”

**DOI:** 10.1155/2018/2718156

**Published:** 2018-09-06

**Authors:** Christophe Milants, Olivier Bruyère, Jean-François Kaux

**Affiliations:** ^1^Physical Medicine, Rehabilitation and Sports Traumatology Department, SportS^2^, FIFA Medical Centre of Excellence, University and University Hospital of Liège, Liège, Belgium; ^2^Department of Public Health, Epidemiology and Health Economics, University of Liège, Liège, Belgium; ^3^Department of Sports and Rehabilitation Sciences, University of Liège, Liège, Belgium

We would like to thank Magalon al. [1] for their constructive comments about our recent publication in Biomed Research International: “Responders to Platelet-Rich Plasma in Osteoarthritis: a technical analysis” [2].

Overall, we agree with most of the different points discussed.

Magalon et al. emphasized the deleterious effects of red blood cells on joints. We strongly support the avoidance of red blood cells in the final platelet-rich plasma (PRP) product used for osteoarthritis or tendinopathy, as already reported several times in our previous publications [3, 4].

In this study, we aimed to assess the PRP formulations, preparation, and uses that would increase the probability of success in the treatment of knee osteoarthritis. This study has been made difficult by the lack, in the published papers, of many information details about the preparation technique and the final PRP product. As Magalon et al. pointed out, the initial platelet count was not mentioned in most of the selected studies. Only an approximation of the platelet count permits the estimate of the total platelet dose injected, which is not supported by evidence based data. We have also underlined many times the advantages of the use of an apheresis machine, which is the only way to have a reproducible PRP including the concentration and number of platelets injected [5].

In their letter, Magalon et al. suggested that “in knee osteoarthritis, more is not necessarily better,” in terms of platelet number. This is, indeed, what this study has pointed out, showing that an intermediate concentration of PRP tended to give the best results [2].

There is no consensus about the ideal concentration and total number of platelets in the PRP product for osteoarthritis. For tendinopathy, a platelet concentration 3 to 4 times that of the whole blood is usually accepted (levels of 600, 000 to 1 000, 000 platelets/*µ*L) [3, 6]. Based on the literature, we agree with the notion of platelet and growth factor doses introduced by Magalon et al. that correspond to the quantity of platelets and growth factor hypothetically delivered at the injection site [7]. It was demonstrated that too concentrated PRP might have a paradoxically inhibitory effect on tissue regeneration [8] and promotes inflammation and collagen deposition [9], supporting, once again, that in knee OA, “more is not necessarily better.” The interest of multiple versus a single injection for tendinopathy and OA is still debated and not well documented in literature on knee OA in comparative studies (1 versus multiple injections). In previous publications on chronic patellar tendinopathies [4, 10, 11], we did not show any benefit in a group of patients who had two injections of PRP versus one single injection. Other recent publications showed opposite results, arguing for multiple injections, for OA and tendinopathy [12–14]. We hope future studies will help find the best combination of PRP formulation and number of injections, including to avoid unuseful multiple injections, and increase of the risk of complications and cost of the treatment [11].

Magalon et al. underlined the increasing clinical and commercial interest in PRP [7]. It should be pointed out that potential competing interests from authors or manufacturers, not always adequately reported in published manuscripts, could lead to a limited reporting of all information needed for the standardization.

At last, Magalon et al. underlined the absence of a widely adopted PRP classification system. Mishra and PAW (acronym of platelets, activation, and white blood cells) classifications were described in this study but remained limited [15, 16]. As already pointed out in our systematic review, we support the view of the authors about the need for an international consensus on the minimal PRP characterization required prior to injection.

In conclusion, step by step, the standardization of PRP therapies should be improved by a better standardization of the PRP products and high level clinical series (RCTs).
